# Comparative- and network-based proteomic analysis of bacterial chondronecrosis with osteomyelitis lesions in broiler’s proximal tibiae identifies new molecular signatures of lameness

**DOI:** 10.1038/s41598-023-33060-y

**Published:** 2023-04-12

**Authors:** Jennifer Cook, Elizabeth S. Greene, Alison Ramser, Garrett Mullenix, Jalila S. Dridi, Rohana Liyanage, Robert Wideman, Sami Dridi

**Affiliations:** 1 Department of Poultry Science, University of Arkansas, 1260 W. Maple Street, Fayetteville, AR 72701 USA; 2 École Universitaire de Kinésithérapie, Université d’Orléans, Rue de Chartres, 45100 Orléans, France

**Keywords:** Molecular biology, Biomarkers

## Abstract

Bacterial Chondronecrosis with Osteomyelitis (BCO) is a specific cause of lameness in commercial fast-growing broiler (meat-type) chickens and represents significant economic, health, and wellbeing burdens. However, the molecular mechanisms underlying the pathogenesis remain poorly understood. This study represents the first comprehensive characterization of the proximal tibia proteome from healthy and BCO chickens. Among a total of 547 proteins identified, 222 were differentially expressed (DE) with 158 up- and 64 down-regulated proteins in tibia of BCO vs. normal chickens. Biological function analysis using Ingenuity Pathways showed that the DE proteins were associated with a variety of diseases including cell death, organismal injury, skeletal and muscular disorder, immunological and inflammatory diseases. Canonical pathway and protein–protein interaction network analysis indicated that these DE proteins were involved in stress response, unfolded protein response, ribosomal protein dysfunction, and actin cytoskeleton signaling. Further, we identified proteins involved in bone resorption (osteoclast-stimulating factor 1, OSFT1) and bone structural integrity (collagen alpha-2 (I) chain, COL2A1), as potential key proteins involved in bone attrition. These results provide new insights by identifying key protein candidates involved in BCO and will have significant impact in understanding BCO pathogenesis.

## Introduction

Bacterial Chondronecrosis with Osteomyelitis (BCO) is the leading cause of lameness affecting tens of billions of broiler chickens worldwide^1,2^. Addition to the food safety issue as well as animal health and welfare concerns, BCO causes heavy annual economic losses to the poultry industries^2^. While BCO likely arises from a complex interaction of metabolic, functional, genetic, nutritional, and environmental factors, there is a clear consensus and strong evidence of bacterial colonization and infection of the proximal femora and tibiae^3,4^.

**Figure 1:**
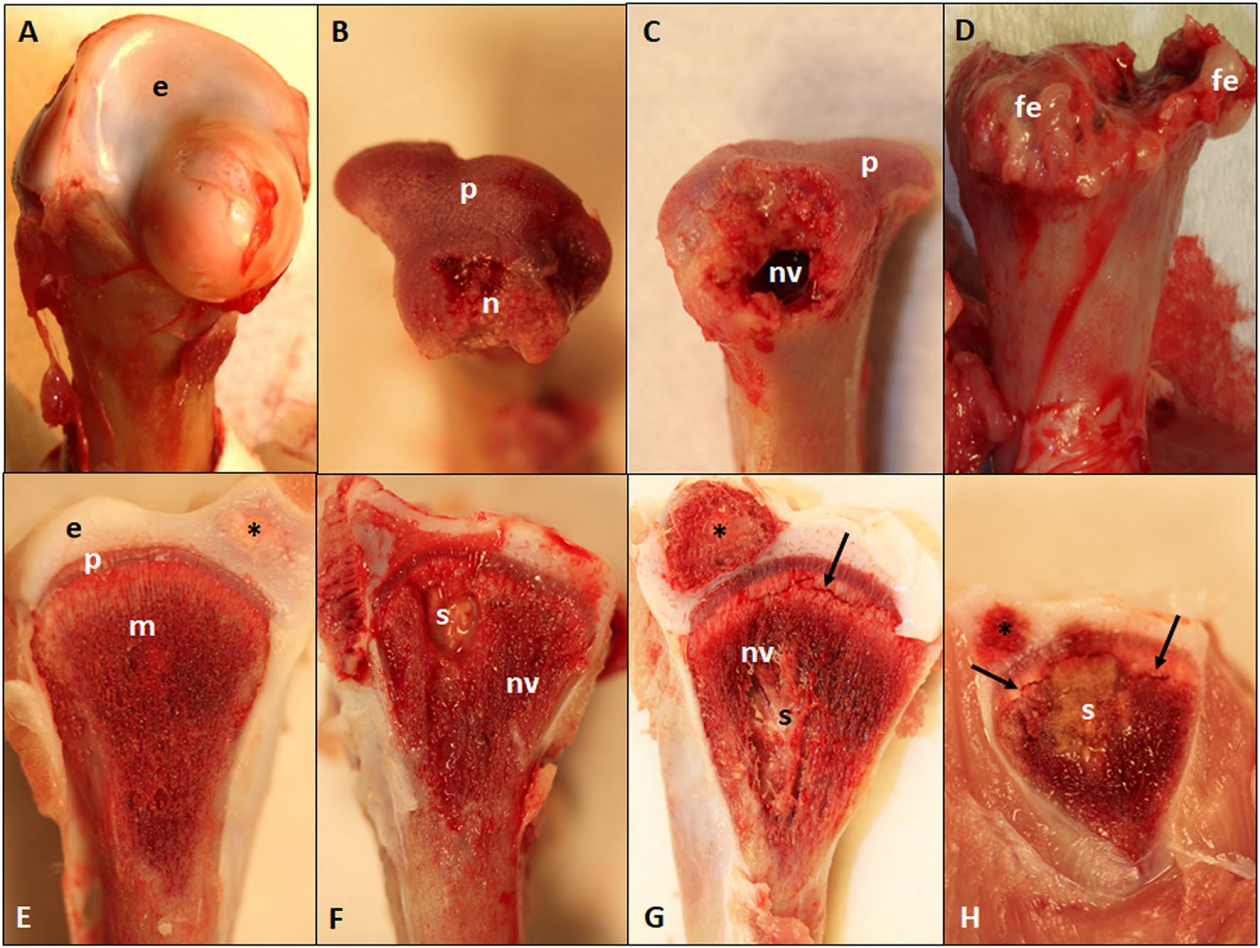
Stages of proximal femoral degeneration (**A**–**D**) and proximal tibial degeneration (**E**–**H**) leading progressively to bacterial chondronecrosis with osteomyelitis: (**A**) Normal proximal femur with white cap of epiphyseal cartilage (**e**); (**B**) Femoral head separation (FHS: epiphyseolysis) with the epiphysis remaining in the socket when the femur was disarticulated, revealing the underlying surface of the growth plate or physis (p) and an early region of necrosis (n); (**C**) Fracturing of the growth plate (p) revealing a necrotic void (nv) within the metaphysis; (**D**) Terminal femoral head necrosis in which the femoral epiphysis, physis and most of the metaphysis remained attached to the acetabulum when the diaphysis weakened by widely dispersed necrosis was fractured during disarticulation, revealing copious fibrinonecrotic exudate (fe); (**E**) Normal proximal tibia showing the epiphysis (e) with a secondary center of ossification (*) and the physis/growth plate (p) fully supported by struts of trabecular bone in the metaphyseal zone (m); (**F**–**H**) Bacterial infiltration and sequestrae (s), necrotic voids (nv) and microfractures below the growth plate (arrows) provide macroscopic evidence of bone damage associated with osteomyelitis.

Multiple opportunistic organisms have been reported from BCO lesions, including *Staphylococcus aureus*, *Staphylococcus agnetis, E. coli*, and *Enterococcus cecorum*, often in mixed cultures with other bacteria^3,5,6^. Bacteria transmitted to chicks from breeder parents, contaminated eggshells or hatchery sources^6,7^**,** or that enter the chick's circulation via translocation through the integument, respiratory system or gastrointestinal tract^8^ spread hematogenously and exit the bloodstream through the capillaries supplying the growth plate. The translocated bacteria bind to the bone collagen and adhere directly to the cartilage matrix, colonize osteochondrotic clefts and form obstructive emboli in the metaphyseal vasculature^9^. Lytic substances released at sites of bacterial colonization promote generalized necrosis within the calcifying zone of the metaphysis, destroying the vasculature and eliminating struts of trabecular bone that normally provide structural support to prevent micro-fracturing of the epiphyseal and physeal cartilage^10,11^. Terminal BCO presents as necrotic degeneration and bacterial infection primarily within the proximal head of the femora and tibiae (Fig. 1), as well as in the growth plates of the 4^th^ thoracic vertebrae.

Despite seminal nutritional and managerial efforts to reduce BCO incidence in chickens, insufficient progress has been made due to limited understanding of the molecular mechanisms underlying BCO pathogenesis. We, therefore, undertook this study using high-throughput proteomics approach combined with advanced bioinformatics to identify molecular signatures of BCO disorder.

## Materials and methods

### Ethics statement

The present study was conducted in accordance with the recommendations in the guide for the care and use of laboratory animals of the National Institutes of Health. All procedures of animal care compiled with and were approved by the University of Arkansas Animal Care and Use Committee (IACUC) under protocol N^o^ 15043. All reported methods are in accordance with the Animal Research Reporting of in Vivo Experiments (ARRIVE).

### Animals and samples preparation

The experiment was conducted at the Poultry Environmental Research Laboratory at the University of Arkansas Poultry Research Farm. One day-old male boiler chicks were obtained from a local commercial hatchery (Cobb-Vantress, Siloam Springs, AR) and randomly divided into two body weight-matched groups. The control group was reared on clean shaved wood litters at 50 birds/pen (6 pens/group, 300 birds/group) and the BCO group was maintained on wire floor model developed by Wideman Robert^10^. Pen conformation, bird densities, diet and water (ad libitum), light/dark cycle, and heating conditions were as previously described^10,12^. To minimize distress, birds were walked on a daily basis from day 15 to the end of the experiment (day 56). On day 56, birds were humanely euthanized, necropsied, and the right and left tibia were macroscopically scored for tibial head necrosis (THN) severity. Briefly, as described previously^13^, THN lesion severity was scored on a 0- to 3-scale with the following categories: 0- no abnormalities (Normal); 1- mild necrosis (THN); 2- severe tibial head necrosis (THNS); and 3- caseous THN (THNC).

### Tibia samples and protein extraction

Chicken tibia samples were collected from healthy (score 0) and BCO (score 3) chickens (n = 6/group), snap frozen in liquid nitrogen and stored at −80 °C until use. Tibia proteins were extracted as previously described^14^. Briefly, samples were homogenized in lysis buffer (10 mM Tris base, pH 7.4, 150 mM NaCl, 1 mM EDTA, 1 mM EGTA, 0.1% Triton X-100, 0.5% NP-40, protease, and phosphatase inhibitors), and proteins (100 μg) were run on 4–12% Novex Bis–Tris gels (ThermoFisher Scientific, Waltham, MA). The gel was then stained with Coomassie blue dye, and de-stained until the background was clear. Gel portions of each sample were excised and chopped into small pieces (< 1 mm^2^) and washed twice with 25 mM NH4HCO3 (Sigma Aldrich, St. Louis, MO). The gel pieces were de-stained with 25 mM NH4HCO3/50% acetonitrile (ACN), and dried with 100% ACN. Proteins were then reduced using 10 mM dithiothreitol (DTT, Sigma Aldrich, St. Louis, MO) in 25 mM NH4CO3 at 56° for 1 h. Subsequently, alkylation was conducted using 55 mM iodoacetamide (IAA, Sigma Aldrich, St. Louis, MO) in 25 mM NH4CO3, protected from light. The gel pieces were then washed with 25 mM NH4HCO3, dehydrated with 25 mM NH4HCO3/50% ACN, and completely dried via SpeedVac. Mass spectrometry grade Trypsin Gold (12.5 ng/μl in 25 mM NH4HCO3, Promega, Madison, WI) was added to cover dried gels, and incubated overnight at 37 °C. Peptides were extracted by 50% ACN/5% formic acid. The tryptic digests were desalted using Pierce C18 spin columns (150 × 0.3 mm, 3.5 µm particle size, 300 Å pore size, ThermoFisher Scientific, Waltham, MA) prior to LC–MS/MS at the State Wide Mass Spectrometry Facility, University of Arkansas at Fayetteville, Arkansas.

### Shotgun proteomics

Individual extracted proteins were used in shotgun proteomics analysis with in-gel trypsin digestion followed by Liquid Chromatography with Tandem Mass Spectrometry (LC–MS/MS) conducted at the State Wide Mass Spectrometry Facility, University of Arkansas at Fayetteville. All LC–MS/MS samples were analyzed using Mascot ((Matrix Science, London, UK; version 2.2.1). Mascot was set up to search the UniProt_Gallus database assuming the digestion enzyme trypsin. Scaffold (version Sacffold_4.8.3, Proteome Software Inc., Portland, OR) was used to validate MS/MS based peptide and protein identifications. Peptide identifications were accepted if they could be established at greater than 95.0% probability by the Scaffold Local FDR algorithm. Protein identifications were accepted if they could be established at > 95.0% probability and contained at least 2 identified peptides. Protein probabilities were assigned by the Protein Prophet algorithm^15,16^. Proteins were annotated with the Gene Ontology Consortium (GO) terms from NCBI^16^. For quantitative changes, we set a *P*-value (*t*-test) < 0.05, set normal as the reference category for fold change and performed total spectra normalization (minimum value 0). Protein–protein interaction analysis was performance by String v10.8 (http://www.string-db.org/) at a 0.4 medium confidence value^17^.

### Bioinformatics and ingenuity pathway analysis

Fold changes of the identified proteins were calculated by comparing the BCO conditions to healthy birds (control) and differentially expressed (DE) proteins (fold change and *P*-value < 0.05) as well as their ID (UniProt^18^) were submitted to QIAGEN Ingenuity Pathway Analysis (IPA, Ingenuity Systems, Redwood City, CA) for core analysis^19^. These proteins were mapped to the most significant canonical pathways, functional annotation, upstream regulators, as well as molecular discovery using the Ingenuity Knowledge Base as a reference set and a cut-off of FDR adjusted *P*-value < 0.05 and a fold-change between -1.5 and 1.5. Right-tailed Fisher’s exact test was used to determine the probability that biological functions and/or diseases were over-represented in the protein dataset. IPA also predicted potential upstream regulators and downstream effectors of the proteins in this study, which were assigned as inhibited or activated according to Z-score^20^.

### Western blot analysis

Proteins were extracted from proximal tibiae tissues as described above and the concentrations were determined using Synergy HT multi-mode microplate reader (BioTek, Winooski, VT) and a Bradford assay kit (Bio-Rad, Hercules, CA) with bovine serum albumin as a standard. Western blot was performed as previously described^14,21^. Briefly, total proteins (100 μg) were resolved on 4–12% Novex Bis–Tris gels (Life Technologies, grand Island, NY), and transferred to a polyvinylidene difluoride (PVDF) membrane. The transferred membranes were blocked in Tris-buffered saline (TBS) with 5% non-fat milk and Tween 20 (TBST) for 1 h at room temperature, and incubated overnight at 4 °C with primary antibodies diluted to 1:500–1:1000. The rabbit polyclonal antibodies used were as follows: anti-HSP90 (cat#PA5-17,610, ThermoFisher Scientific Waltham, MA), anti-OSTF1 (cat#A303-004A, Bethyl Laboratories, Inc, Waltham, MA), anti-ACLY (cat# LS-C290517, Lifespan Biosciences, Seattle, WA), anti-vinculin (VCL, cat#V4139, Sigma-Aldrich, Inc, St. Louis, MO), anti-STAT3 (cat# 4904, Cell Signaling Technology, Danvers, MA), anti-ACTN (cat#A1160, ABClonal Technology, Woburn, MA), and anti-β actin (cat#4967, Cell Signaling Technology, Danvers, MA). The membrane was washed twice with PBS-T and then incubated with anti-mouse or anti-rabbit horseradish peroxidase conjugated secondary antibodies (1:5000) for 1 h at room temperature. The signal was visualized by enhanced chemiluminescence (ECL plus) (GE Healthcare Bio-Sciences, Buckinghamshire, UK) and captured by FluorChem M MultiFluor System (Proteinsimple, Santa Clara, CA). Image Acquisition and Analysis were performed by AlphaView software (Version 3.4.0, 1993–2011, Proteinsimple, Santa Clara, CA).

### Total RNA extraction and real-time quantitative PCR

Total RNA was extracted using Trizol reagent (Life Technologies, Carlsbad, CA) according to the manufacturer’s recommendations, and concentration and quality were determined with the Take3 microvolume plate and the Synergy HT multimode microplate reader (BioTek, Winooski, VT). RT and qPCR were performed as previously described^22^. Briefly, RNA was reverse transcribed using qScript cDNA Synthesis Supermix (Quanta Biosciences, Gaithersburg, MD), and amplified by qPCR (Applied Biosystems 7500 Real Time System) with Power-Up Sybr green master mix (Life Technologies, Carlsbad, CA). Relative expression of the target genes was determined using the 2^-ΔΔCT^ method^23^, with normalization to ribosomal 18S expression and healthy bird as a calibrator (control). Oligonucleotide primer sequences specific for chicken are presented in Table 1.

**Table 1:** Oligonucleotide QPCR primers.

Gene^a^	Accession number^b^	Primer sequence (5′ → 3′)	Orientation	Product size (bp)
*NRF1*	NM_001030646	GGCCAACGTCCGAAGTGAT	Forward	55
		CCATGACACCCGCTGCTT	Reverse	
*Col2A1*	NM_204426	ACCTGCCGCGACATCAA	Forward	60
		GTCAATCCAGTAATCTCCGCTCTT	Reverse	
*HSP90*	X07265	TGACCTTGTCAACAATCTTGGTACTAT	Forward	68
		CCTGCAGTGCTTCCATGAAA	Reverse	
*MYC*	NM_001030952	CCCGAGGTGGCCAACA	Forward	57
		CGTGGCTTTTTTCAGGATGAC	Reverse	
*PR*	NM_205262	CTGGTGAAGGCCATTGGTTT	Forward	61
		GATAGAAGCGCTGGGAGTTAGC	Reverse	
*NRF2*	NM_205117	AAACGACAACCTGGCTGAAGTAA	Forward	59
		TCTCCGCTGGCTTGGTTTC	Reverse	
*NCAM1*	NM_001128828	GAGCACACCGCCGATACTG	Forward	67
		TAGTGGTGCCCGTAGTTACAGAAG	Reverse	
*ACTN4*	NM_001396037	TGGGCATGATCTGGACCAT	Forward	72
		CCTTGGCCGAGGTCTCTTC	Reverse	
*18S*	AF173612	TCCCCTCCCGTTACTTGGAT	Forward	60
		GCGCTCGTCGGCATGTA	Reverse	

^a^ACTN4, α-actinin 4; Col2A1, collagen type II alpha 1 chain; HSP90, heat shock protein 90; MYC, myelocytomatosis oncogene; NCAM1, neural cell adhesion molecule 1; NRF1/2, NFE2 like bZIP transcription factor ½; PR, progesterone receptor.

^b^Accession number refer to Genbank (NCBI).

### Statistical analysis

Data were analyzed using Student “*t*” test and Graph Pad Prism software (version 7.03 for Windows, Graph Pad Software, La Jolla California, USA). Data are expressed as the mean ± SEM. Means were considered statistically significant at a *P* value ≤ 0.05.

## Results

### Protein expression profile in proximal tibiae of healthy- and BCO-affected broilers

To gain further insights into the molecular pathogenesis of BCO, we performed LC–MS/MS on protein isolated from the proximal end of the tibia from control (score 0) and BCO birds with severe THN (score 3). MASCOT and Scaffold analysis identified a total of 547 proteins that have been submitted to EMBL-EBI PRIDE database (https://doi.org/10.6019/PXD029085, accession# PXD029085PXD029085, reference #1-20211011-22240). Quantitative analysis identified 222 differentially expressed (DE) proteins (*T*-test, *P* $<$ 0.05). Of those, there were 158 up- and 64 down-regulated proteins in tibia of BCO *vs.* normal chickens (Tables 2 and 3).

**Table 2:** Differentially expressed (up regulated) proteins in tibia of BCO compared to healthy chickens.

Symbol	Entrez gene name	ID	Exp log change	Expr *p*-value
A2ML1	Alpha-2-macroglobulin like 1	F1NK40	2.994	1.78E-08
ACLY	ATP citrate lyase	Q5F3V2	1.278	0.00204
ACO1	Aconitase 1	ACOC	2.188	0.00081
ACO2	Aconitase 2	Q5ZMW1	1.089	0.0287
ACTA1	Actin, alpha 1, skeletal Muscle	ACTS	1.624	0.00109
ACTG1	Actin gamma 1	ACT5	1.728	0.00136
ACTN1	Actinin alpha 1	ACTN1	1.45	0.000637
AK2	Adenylate kinase 2	F1NJ73	1.429	0.045
Akr1b7	Aldo–keto reductase family 1, member B7	E1C1I6	1.104	0.045
AKR7A2	Aldo–keto reductase family 7 member A2	F1P331	1.061	0.0195
ALB	Albumin	ALBU	0.847	0.0425
ANXA1	Annexin A1	Q6QAZ9	1.122	0.0119
ANXA2	Annexin A2	ANXA2	0.787	0.0193
ANXA5	Annexin A5	ANXA5	1.572	0.0202
AP3B1	Adaptor related protein complex 3 beta 1 subunit	E1BW97	2.142	0.000802
APEX1	Apurinic/apyrimidinic endodeoxyribonuclease 1	B3Y932	1.681	0.0187
ARHGDIA	Rho GDP dissociation inhibitor alpha	Q5ZMT1	1.382	0.00796
ARPC1B	Actin related protein 2/3complex subunit 1B	Q5ZI99	1.398	0.00936
ATIC	5-aminoimidazole-4-carboxamide ribonucleotide formyltransferase/IMP cyclohydrolase	PUR9	1.488	0.0028
ATP1A1	ATPase Na + /K + transporting subunit alpha 1	AT1A1	1.82	0.00093
ATP1A2	ATPase Na + /K + transporting subunit alpha 2	AT1A2	2.323	0.00678
ATP5A1	ATP synthase, H + transporting, mitochondrial F1 complex, alpha subunit 1, cardiac muscle	Q90VX2	1.521	0.0142
ATP6V0D1	ATPase H + transporting V0 subunit d1	E1BVF8	2.795	0.00256
ATP6V0D2	ATPase H + transporting V0 subunit d2	VA0D2	1.524	0.0374
ATP6V1A	ATPase H + transporting V1 subunit A	VATA	1.205	0.044
C6	Complement C6	B8ZX71	1.223	0.0142
C7	Complement C7	E1C6U2	1.715	0.0194
CANX	Calnexin	Q5ZMF5	1.438	0.000614
CBR1	Carbonyl reductase 1	Q4JK63	1.567	0.0107
CDV3	CDV3 homolog	CDV3	1.681	0.0072
CFH	Complement factor H	E1C7P4	0.983	0.0125
CLIC2	Chloride intracellular Channel 2	Q5ZKI1	1.63	0.00109
CLTC	Clathrin heavy chain	Q8UUR0	1.453	0.0216
COPB2	Coatomer protein complex subunit beta 2	E1C016	1.687	0.0256
COPE	Coatomer protein complex subunit epsilon	COPE	2.067	0.00399
COPG1	Coatomer protein complex subunit gamma 1	F1NB52	1.312	0.00405
CTSS	Cathepsin S	H9KYW5	1.658	0.00958
CTSZ	Cathepsin Z	E1C4M3	1.841	0.0227
ECHS1	Enoyl-CoA hydratase, short chain 1	F1NR44	1.634	0.0221
EEA1	Early endosome antigen 1	E1BRE5	1.428	0.013
EEF1A1	Eukaryotic translation Elongation factor 1 alpha 1	EF1A	1.9	0.00482
EEF2	Eukaryotic translation elongation factor 2	EF2	1.477	0.0000391
EIF2S1	Eukaryotic translation initiation factor 2 subunit Alpha	IF2A	1.281	0.0347
ENO1	Enolase 1	ENOA	1.601	0.014
ETFA	Electron transfer flavoprotein alpha subunit	F1N9U8	0.881	0.0384
FDPS	farnesyl diphosphate Synthase	FPPS	1.657	0.018
FGA	Fibrinogen alpha chain	FIBA	2.299	0.00246
FGB	Fibrinogen beta chain	FIBB	1.846	0.0293
FKBP3	FK506 binding protein 3	Q90ZK7	1.73	0.0343
FLNA	Filamin A	Q90576	2.422	0.0222
FLNB	Filamin B	F1N8D4	1.076	0.008
FLNC	Filamin C	Q90WF0	1.939	0.0439
FN1	Fibronectin 1	FINC	0.91	0.0273
GAPDH	Glyceraldehyde-3-Phosphate dehydrogenase	G3P	1.379	0.000447
GBP2	Guanylate binding protein 2	Q90892	0.989	0.0383
GCLM	Glutamate-cysteine ligase modifier subunit	Q5ZL03	2.308	0.00316
GNAO1	G protein subunit alpha o1	E1C347	1.101	0.0437
GPI	Glucose-6-phosphate isomerase	Q5ZMU3	1.746	0.0137
HLA-DPB1	Major histocompatibility complex, class II, DP beta 1	HB2L	3.014	0.00389
HMGB1	High mobility group box 1	HMGB1	2.663	0.0029
HMGB3	High mobility group box 3	HMGB3	3.114	0.00153
HMOX1	Heme oxygenase 1	HMOX1	1.82	0.00505
HNRNPA1	Heterogeneous nuclear ribonucleoprotein A1	E1BZE6	1.738	0.00571
HNRNPH1	Heterogeneous nuclear ribonucleoprotein H1	Q5F3I8	1.666	0.00979
HPX	Hemopexin	H9L385	2.04	0.0000274
HSP90AA1	Heat shock protein 90 alpha family class A member 1	HS90A	3.182	4.33E-09
HSP90AB1	Heat shock protein 90 alpha family class B member 1	HS90B	2.471	0.00279
HSP90B1	Heat shock protein 90 beta family member 1	ENPL	2.595	6.46E-08
HSPD1	Heat shock protein family D (Hsp60) member 1	CH60	1.404	0.000777
IFI30	IFI30, lysosomal thiol reductase	K7XM59	2.694	0.000609
IPO7	importin 7	F1NBA8	1.523	0.000367
IQGAP2	IQ motif containing GTPase activating protein 2	E1C2F9	0.967	0.0208
ITGAV	Integrin subunit alpha V	F1NGX1	2.627	0.00099
ITGB1	Integrin subunit beta 1	ITB1	1.154	0.0328
KRT7	Keratin 7	K2CO	1.803	0.00479
KRT75	Keratin 75	Q6PVZ3	1.415	0.00923
Ktn1	Kinectin 1	KTN1	1.318	0.0269
L1CAM	L1 cell adhesion molecule	NGCA	1.834	0.0138
LARP1B	La ribonucleoprotein Domain family member 1B	F1NBQ1	2.496	0.00118
LCP1	Lymphocyte cytosolic protein 1	Q5ZLW0	1.258	0.00308
LDHA	Lactate dehydrogenase A	LDHA	1.075	0.00476
LECT2	leukocyte cell derived chemotaxin 2	MIM1	1.228	0.0105
MTAP	Methylthioadenosine phosphorylase	Q5ZHQ7	1.599	0.00131
MYH10	Myosin heavy chain 10	Q789A6	0.911	0.0158
MYH11	Myosin heavy chain 11	MYH11	1.056	0.0186
MYH9	Myosin heavy chain 9	MYH9	0.839	0.0058
NAP1L1	Nucleosome assembly Protein 1 like 1	E1BZS2	1.389	0.0308
NDUFA10	NADH:ubiquinone oxidoreductase subunit A10	F1NPM6	1.178	0.0193
NDUFA9	NADH:ubiquinone oxidoreductase subunit A9	Q5ZI00	2.252	0.0196
NEB	nebulin	Q9DEH4	2.343	0.0432
NNT	Nicotinamide nucleotide transhydrogenase	E1C6A1	1.254	0.042
NONO	Non-POU domain containing octamer binding	Q5ZIZ5	2.836	0.000753
NPM1	nucleophosmin 1	NPM	2.157	0.000421
NUBP2	Nucleotide binding protein 2	NUBP2	1.903	0.00403
OSTF1	Osteoclast stimulating factor 1	OSTF1	2.05	0.000569
P4HA1	Prolyl 4-hydroxylase subunit alpha 1	P4HA1	1.35	0.0221
P4HB	Prolyl 4-hydroxylase subunit beta	PDIA1	1.283	0.000364
PCBP3	Poly(rC) binding protein 3	E1BVU6	1.923	0.0231
PDIA3	Protein disulfide isomerase family A member 3	PDIA3	1.015	0.0076
PGAM1	phosphoglycerate mutase 1	PGAM1	1.549	0.00109
PGLS	6-Phosphogluconolactonase	Q5ZJY1	1.912	0.00916
PGM1	Phosphoglucomutase 1	F1NN63	2.365	0.00338
PGM2	Phosphoglucomutase 2	Q5ZHU2	1.861	0.00829
PHB	Prohibitin	PHB	1.392	0.0012
PKM	Pyruvate kinase M1/2	F1NW43	1.573	0.00384
PLS3	Plastin 3	Q5ZI39	2.617	0.00659
PPP1CB	Protein phosphatase 1 Catalytic subunit beta	PP1B	1.056	0.0213
PRKAR1A	Protein kinase cAMP-Dependent type I Regulatory subunit alpha	KAP0	1.503	0.0407
PSMA6	Proteasome subunit alpha 6	F1NEQ6	2.406	0.0118
PSMA7	Proteasome subunit alpha 7	PSA7	1.057	0.0403
PSMD1	Proteasome 26S subunit, Non-ATPase 1	PSMD1	1.777	0.0303
PTPA	Protein phosphatase 2 Phosphatase activator	Q5ZM56	2.049	0.0169
PTPRC	Protein tyrosine Phosphatase, receptor type C	Q91976	1.079	0.0148
RAN	RAN, member RAS oncogene family	RAN	1.192	0.00915
RDX	radixin	RADI	1.975	0.0287
RPL19	Ribosomal protein L19	Q5ZKK8	1.518	0.0247
RPL6	Ribosomal protein L6	Q8UWG7	1.536	0.0133
RPL7A	Ribosomal protein L7a	RL7A	2.143	0.00127
RPLP0	Ribosomal protein lateral Stalk subunit P0	RLA0	1.178	0.0056
RPRD1B	Regulation of nuclear pre-mRNA domain containing 1B	Q5F4B4	1.627	0.0217
RPS2	Ribosomal protein S2	E1C4M0	1.057	0.00802
RPS3	Ribosomal protein S3	F1NPA9	0.9	0.0242
Rps3a1	Ribosomal protein S3A1	F2Z4K7	2.126	0.0125
RPSA	Ribosomal protein SA	RSSA	1.099	0.0315
Rrbp1	Ribosome binding protein 1	F1P0K1	0.81	0.0381
RTRAF	RNA transcription, translation and transport factor	Q90706	1.469	0.0303
S100A9	S100 calcium binding protein A9	M126	1.732	0.00617
SDHB	Succinate dehydrogenase complex iron sulfur subunit B	SDHB	2.214	0.00434
SET	SET nuclear proto-oncogene	F2Z4L4	2.014	0.0000528
SFPQ	Splicing factor proline and glutamine rich	F1P555	2.303	0.00396
SPINK5	Serine peptidase inhibitor, Kazal type 5	IOV7	2.543	0.00707
SSR1	Signal sequence receptor subunit 1	Q5ZM63	1.319	0.0123
STAT1	Signal transducer and Activator of transcription 1	Q5ZJK3	2.184	0.00508
STOM	stomatin	E1BTV1	1.506	0.00275
STRAP	SERINE/threonine kinase receptor associated protein	STRAP	1.507	0.023
TALDO1	transaldolase 1	Q5ZKN8	0.962	0.0147
TARS	Threonyl-tRNA synthetase	Q5ZLW1	1.662	0.00576
TFRC	Transferrin receptor	TFR1	1.583	0.000467
TKT	Transketolase	F1P1A5	1.553	0.00515
TLN1	Talin 1	TLN1	0.696	0.0314
TPI1	Triosephosphate isomerase 1	TPIS	1.378	0.00227
TTR	Transthyretin	TTHY	1.001	0.0459
UCHL3	Ubiquitin C-terminal Hydrolase L3	Q9PW67	2.033	0.00207
UGGT1	UDP-glucose glycoprotein glucosyltransferase 1	F1NTV6	0.98	0.00462
UGGT2	UDP-glucose glycoprotein glucosyltransferase 2	E1BQH9	1.242	0.0278
USP9X	Ubiquitin specific peptidase 9, X-linked	V9GVG9	5.198	0.0000279
VAPA	VAMP associated protein A	Q5F419	1.213	0.0275
VAPB	VAMP associated protein B and C	F1NSW0	2.802	0.00327
VCL	Vinculin	VINC	1.139	0.00844
VCP	Valosin containing protein	Q5ZMU9	1.343	0.00137
VDAC2	Voltage dependent anion channel 2	Q9I9D1	2.042	0.000333
VNN1	Vanin 1	Q5ZHM4	2.134	0.000352
VPS35	VPS35, retromer complex component	F1NVF0	1.074	0.0133
VTN	Vitronectin	O12945	1.427	0.00508
YWHAB	tyrosine 3-Monooxygenase/tryptophan 5-monooxygenase activation protein beta	1433B	0.937	0.0101
YWHAG	tyrosine 3-monooxygenase/tryptophan 5-monooxygenase activation protein gamma	F1NMY1	2.408	0.0127
YWHAQ	tyrosine 3-monooxygenase/tryptophan 5-monooxygenase activation protein theta	1433 T	1.524	0.000826
YWHAZ	Tyrosine 3-monooxygenase/tryptophan 5-monooxygenase activation protein zeta	1433Z	1.537	0.00203

**Table 3:** Differentially expressed (down regulated) proteins in tibia of BCO compared to healthy chickens.

Symbol	Entrez gene name	ID	Expr log ratio	Expr p-value
ACTN4	actinin alpha 4	ACTN4	− 3.062	0.00108
AKR1D1	aldo–keto reductase family 1 member D1	E1BU27	− 1.121	0.0235
ALDOC	aldolase, fructose-bisphosphate C	R4GM10	− 1.85	0.00843
APOA1	apolipoprotein A1	APOA1	− 1.632	0.0173
APOA4	apolipoprotein A4	O93601	− 1.394	0.00197
ATP6V1D	ATPase H + transporting V1 subunit D	E1BT00	− 1.467	0.00634
ATP6V1E1	ATPase H + transporting V1 subunit E1	Q5ZKJ9	− 1.406	0.00705
C3	complement C3	A6N9E0	− 1.802	0.00125
C4BPA	complement component 4 binding protein alpha	Q4AEJ1	− 0.962	0.0337
C9orf64	chromosome 9 open reading frame 64	E1C455	− 2.699	0.00741
CCDC93	coiled-coil domain containing 93	CCD93	− 3.929	0.0408
CCT2	chaperonin containing TCP1 subunit 2	Q5F424	− 1.309	0.00143
CCT5	chaperonin containing TCP1 subunit 5	Q5F411	− 1.198	0.0324
CCT7	chaperonin containing TCP1 subunit 7	TCPH	− 1.306	0.0124
CHMP2A	charged multivesicular body protein 2A	CHM2A	− 2.346	0.00683
CIAPIN1	cytokine induced apoptosis inhibitor 1	CPIN1	− 1.464	0.0089
CKM	creatine kinase, M-type	KCRM	− 3.636	0.0219
COL2A1	collagen type II alpha 1 chain	Q90W37	− 3.141	0.0288
COPG2	coatomer protein complex subunit gamma 2	E1C7T8	− 2.588	0.0147
DCN	decorin	PGS2	− 1.447	0.0206
ELAVL1	ELAV like RNA binding protein 1	Q9PW24	− 1.175	0.0421
EMC1	ER membrane protein complex subunit 1	EMC1	− 1.454	0.0367
EPYC	epiphycan	EPYC	− 1.79	0.043
ERP29	endoplasmic reticulum protein 29	F1NRM8	− 0.953	0.0084
F13A1	coagulation factor XIII A chain	E1BWG1	− 2.103	0.00646
GLS	glutaminase	Q5ZIV6	− 3.96	0.0149
GNAQ	G protein subunit alpha q	Q5F3B5	− 2.009	0.00438
Gstt3	glutathione S-transferase, theta 3	E1BUB6	− 2.205	0.00202
HADHA	hydroxyacyl-CoA dehydrogenase trifunctional multienzyme complex subunit alpha	F1NI29	− 1.14	0.0189
HBE1	hemoglobin subunit epsilon 1	HBB	− 0.949	0.0301
IMMT	inner membrane mitochondrial protein	F1P3U1	− 1.228	0.0231
ITIH3	inter-alpha-trypsin inhibitor heavy chain 3	F1NIE3	− 0.906	0.0278
LAMP1	lysosomal associated membrane protein 1	LAMP1	− 2.093	0.00488
LMNB2	lamin B2	LMNB2	− 0.814	0.02
LUM	lumican	LUM	− 1.621	0.0412
MBD5	methyl-CpG binding domain protein 5	F1NE18	− 2.729	0.00309
MDH1	malate dehydrogenase 1	MDHC	− 1.15	0.0202
MDH2	malate dehydrogenase 2	E1BVT3	− 0.913	0.0243
MYOF	myoferlin	E1BW21	− 2.236	0.00781
NCAM1	neural cell adhesion molecule 1	F1NYN0	− 3.868	0.0207
NUCB2	nucleobindin 2	Q5ZHR1	− 1.216	0.00561
PAPSS2	3'-phosphoadenosine 5'-phosphosulfate synthase 2	F1NPR8	− 1.369	0.0334
PCNA	proliferating cell nuclear antigen	PCNA	− 1.621	0.0218
PDLIM1	PDZ and LIM domain 1	E1C852	− 2.004	0.00638
PRDX6	peroxiredoxin 6	PRDX6	− 1.318	0.0253
PSMD14	proteasome 26S subunit, non-ATPase 14	E1C6N0	− 2.06	0.00419
PSME4	proteasome activator subunit 4	R4GFD0	− 1.691	0.0199
PTBP1	polypyrimidine tract binding protein 1	Q5F456	− 1.459	0.00538
RPN1	ribophorin I	E1C0F1	− 1.245	0.0176
SCIN	scinderin	ADSV	− 1.101	0.00953
SEC24D	SEC24 homolog D, COPII coat complex component	E1BSP8	− 2.227	0.0416
SEC61A1	Sec61 translocon alpha 1 subunit	F1NBN1	− 2.309	0.00451
SLC25A4	solute carrier family 25 member 4	Q5ZMJ6	− 0.924	0.0137
SLC4A1	solute carrier family 4 member 1 (Diego blood group)	B3AT	− 1.526	0.0275
SNRPA1	small nuclear ribonucleoprotein polypeptide A'	Q5ZKP1	− 1.167	0.0466
SPTAN1	spectrin alpha, non-erythrocytic 1	F1NHT3	− 1.929	0.00262
STAT3	signal transducer and activator of transcription 3	STAT3	− 3.58	0.00102
SUPT6H	SPT6 homolog, histone chaperone	E1C1S5	− 2.188	0.0191
TAP2	transporter 2, ATP binding cassette subfamily B member	B5BSL8	− 1.504	0.0496
TCP1	t-complex 1	Q5ZMG9	− 0.906	0.0157
TF	transferrin	E1BQC2	− 4.024	0.0179
TPM1	tropomyosin 1	TPM1	− 1.247	0.0498
Tpm3	tropomyosin 3	Q5ZLJ7	− 2.211	0.0036
UPF1	UPF1, RNA helicase and ATPase	E1C0J4	− 2.67	0.0101

### Metabolic pathway and network analysis

To gain biologically related molecular networks, the above identified DE proteins (222) were submitted into IPA knowledge-base. From these 222 proteins, 153 IDs were successfully mapped, while 69 IDs were unmapped to molecules in the Ingenuity Knowledge Base. The 153 mapped proteins were analyzed to outline the most enriched biological functions.

As shown in Table 4, the top five canonical pathways associated with Huntington’s disease, inhibition of ARE-mediated mRNA degradation, protein ubiquitination, hepatic fibrosis signaling, and glycolysis were enriched in BCO THN.

**Table 4:** Top canonical pathways enriched by observed protein alteration in BCO tibiae.

Canonical pathways	Proteins^1^	*P*-Value	Ratio	Overlap
Huntington’s disease signaling	GLS, GNAQ, PSMA6, PSMD1, PSMD14, PSME4, SDHB	1.23 10^–2^	1	100%
Inhibition of ARE-mediated mRNA degradation pathway	PSMA6, PSMD1, PSMD14, PSME4, PTPA, YWHAG	2.34 10^–2^	1	100%
Protein ubiquitination pathway	PSMA6, PSMD1, PSMD14, TAP2, UCHL3	4.44 10^–2^	1	100%
Hepatic fibrosis signaling pathway	COL2A1, ITGAV, SDHB, STAT3, TF	4.44 10^–2^	1	100%
Glycolysis	ALDOC, GPI, PGAM1, PKM	8.37 10^–2^	1	100%

^1^ALDOC, Aldolase, fructose-bisphosphate C; GLS, Glutaminase; GNAQ, G protein subunit alpha q; GPI, Glucose-6-phosphate isomerase; PGAM1, Phosphoglycerate mutase 1; PKM, Pyruvate kinase M1/2; PSMA, Proteasome 20S subunit alpha; PSMD, Proteasome 26S subunit, non-ATPase; PSME4, Proteasome activator subunit 4; PTPA, Protein phosphatase 2 phosphatase activator; SDHB, Succinate dehydrogenase complex iron sulfur subunit B; STAT3, Signal transducer and activator of transcription 3; TAP2, Transporter 2, ATP binding cassette subfamily B member; TF, Transferrin; UCHL3, Ubiquitin C-terminal hydrolase L3; YWHAG, Tyrosine 3-monooxygenase/tryptophan 5-monooxygenase activation protein gamma.

The top diseases and disorders enriched by the IPA core analysis for the DE proteins were ranked by *P*-value and summarized in Table 5. Cancer and organismal injury and abnormalities (including chronic bone and joint disease) were the top disorders enriched with 32 and 46 proteins, respectively (*P* = 4.83 × 10^–2^–3.05 × 10^–3^). Next, cardiovascular disease was embellished by IPA analysis and ranked the third with 15 proteins (*P* = 4.44 × 10^–2^-6.65 × 10^–3^), followed by connective tissue disorders and inflammatory disease with 10 molecules each (*P* = 0.01).

**Table 5:** Top diseases and disorders for DE proteins in broiler tibiae with BCO.

Diseases and disorders	*P*-Value range	# Mol	Molecules^1^
Cancer	4.83 10^–2^–3.05 10^–3^	33	APOA1, HMGB1, LUM, CTSS, HMGB3, PKM, EEF1A1, HMOX1, STAT1, GLS, ITGAV, STAT3, COL2A1, TF, GNAQ, SDHB, PCNA, ACTN4, CTSZ, PDLIM1, ANXA5, GPI, MYOF, PLS3, CBR1, PSMD1, UPF1, C7, NDUFA9, RPL6, NONO, UCHL3, HMGB8
Organismal Injury and abnormalities	4.83 10^–2^–3.05 10^–3^	46	APOA1, HMGB1, LUM, CTSS, HMGB3, PKM, EEF1A1, HMOX1, STAT1, GLS, ITGAV, STAT3, COL2A1, TF, GNAQ, SDHB, PCNA, VNN1, F13A1, GCLM, ACTN4, CTSZ, PDLIM1, ANXA5, GPI, MYOF, PLS3, CBR1, PSMD1, UPF1, C7, NDUFA9, RPL6, NONO, PSMD14, VAP8, ATP6V0D1, HNRNPA1, SUPT6H, UCHL3, SPTAN1, TKT, ALDOC, MTAP, APEX1, FLNC
Cardiovascular disease	4.44 10^–2^–6.65 10^–3^	15	APOA1, HMGB1, PKM, VNN1, CTSS, HMOX1, STAT1, F13A1, ITGAV, STAT3, GCLM, PCNA, TF, COL2A1, GNAQ
Connective tissue disorders	1.0 10^–2^	10	APOA1, GPI, STAT1, COL2A1, HMGB1, STAT3, CTSS, HMOX1, F13A1, PKM
Inflammatory disease	1.0 10–2	10	APOA1, GPI, STAT1, COL2A1, HMGB1, STAT3, CTSS, HMOX1, F13A1, PKM

^1^ACTN4, acTinin alpha 4; ALDOC, Aldolase, fructose-bisphosphate C; ANXA5, Annexin A5; APEX1, Apurinic/apyrimidinic endodeoxyribonuclease 1; APOA1, Apolipoprotein A1; ATP6V0D1, ATPase H + transporting V0 subunit d1; C7, Complement C7; CBR1, Carbonyl reductase 1; COL2A1, Collagen type II alpha 1 chain; CTSS, cathepsin S; CTSZ, Cathepsin Z; EEF1A1, Eukaryotic translation elongation factor 1 alpha 1; F13A1, Coagulation factor XIII A chain; FLNC, Filamin C; GCLM, Glutamate-cysteine ligase modifier subunit; GLS, Glutaminase; GNAQ, G protein subunit alpha q; GPI, Glucose-6-phosphate isomerase; HMGB1/3, High mobility group box 1/3; HMOX1, Heme oxygenase 1; HNRNPA1, Heterogeneous nuclear ribonucleoprotein A1, ITGAV, Integrin subunit alpha V; LUM, Lumican; MTAP, Methylthioadenosine phosphorylase; MYOF, Myoferlin; NDUFA9, NADH:Ubiquinone oxidoreductase subunit A9; NONO, Non-POU domain containing octamer binding; PCNA, Proliferating cell nuclear antigen; PDLIM1, PDZ and LIM domain 1; PKM, pyruvate kinase M1/2; PLS3, plastin 3; PSMD, Proteasome 26S subunit, non-ATPase 1; RPL6, Ribosomal protein L6; SDHB, Succinate dehydrogenase complex iron sulfur subunit B; STAT1/3, Signal transducer and activator of transcription 1/3; SPTAN1, Spectrin alpha chain, non-erythrocytic 1; SUPT6H, SPT6 homolog, histone chaperone and transcription elongation factor; TF, transferrin; TKT, Transketolase; UCHL3, Ubiquitin C-terminal hydrolase L3; UPF1, UP-frameshift-1; VAP8, Amyotrophic Lateral Sclerosis 8 protein; VNN1, Vanin 1.

The top five molecular and cellular functions generated by IPA core analysis and rated by *P*- values are cellular development (*P* = 4.4 × 10^–2^–1.7 × 10^–2^, 17 molecules), cellular growth and proliferation (*P* = 4.4 × 10^–2^–1.7 × 10^–2^, 16 molecules), cell cycle (*P* = 3.2 × 10^–2^–3.2 × 10^–2^, 10 molecules), gene and protein expression (*P* = 4.8 × 10^–2^–3.2 × 10^–2^, 17 molecules), and cell-to-cell signaling and interaction (*P* = 4.4 × 10^–2^, 5 molecules) (Table 6).

**Table 6:** Top molecular and cellular functions for DE proteins in broiler tibiae with BCO.

Molecular & cellular functions	*P*-Value	# proteins	Proteins^1^
Cellular Development	4.4 10^–2^–1.7 10^–2^	17	CTSS, HMGB3, STAT3, EEF1A1, LUM, GLS, PKM, HMGB1, STAT1, ACTN4, PCNA, UCHL3, CTSZ, HMOX1, MBD5, APOA1, IPO7
Cellular growth and proliferation	4.4 10^–2^–1.7 10^–2^	16	CTSS, HMGB3, STAT3, EEF1A1, LUM, GLS, PKM, HMGB1, STAT1, ACTN4, PCNA, UCHL3, CTSZ, HMOX1, MBD5, IPO7
Cell cycle	3.2 10^–2^–3.2 10^–2^	10	APEX1, RPRD1B, TF, HMGB1, SET, YWHAG, PCNA, STAT1, PKM, STAT3
Gene/protein expression	4.8 10^–2^–3.2 10^–2^	17	APEX1, RPRD1B, TF, HMGB1, SET, YWHAG, PCNA, STAT1, PKM, STAT3, ACTN4, HNRNPA1, COL2A1, PSMD14, GNAQ, SEC61A1, STRAP
Cell-to-cell signaling and interaction	4.4 10^–2^–4.4 10^–2^	5	APOA1, STAT1, CTSZ, HMGB, PKM

^1^ACTN4, Actinin alpha 4; APEX1, Apurinic/apyrimidinic endodeoxyribonuclease 1; APOA1, Apolipoprotein A1; COL2A1, Collagen type II alpha 1 chain; CTSS, Cathepsin S; CTSZ, Cathepsin Z; EEF1A1, Eukaryotic translation elongation factor 1 alpha 1; GLS, Glutaminase; GNAQ, G protein subunit alpha q; HMGB1/3, High mobility group box 1/3; HMOX1, Heme oxygenase 1; HNRNPA1, Heterogeneous nuclear ribonucleoprotein A1; IPO7, Importin 7; LUM, lumican; MBD5, Methyl-CpG binding domain protein 5; PCNA, Proliferating cell nuclear antigen; PKM, Pyruvate kinase M1/2; PSMD14, Proteasome 26S subunit, non-ATPase 14; RPRD1B, Regulation of nuclear pre-mRNA domain containing 1B; SEC61A1, SEC61 translocon subunit alpha 1; SET, SET nuclear proto-oncogene; STAT1/3, Signal transducer and activator of transcription 1/3; STRAP, Serine/threonine kinase receptor associated protein; TF, Transferrin; UCHL3, Ubiquitin C-terminal hydrolase L3; YWHAG, Tyrosine 3-monooxygenase/tryptophan 5-monooxygenase activation protein gamma.

The top physiological system development and functions enriched by IPA are summarized in Table 7 and are composed of organismal survival (*P* = 0.03, 26 molecules), connective tissue development and function (*P* = 0.04, 5 proteins), hematological system development and function (*P* = 0.04, 9 molecules), hematopoiesis (*P* = 0.04, 5 molecules), and immune cell trafficking (*P* = 0.04, 5 molecules).

**Table 7:** Top physiological system development and function for DE proteins in broiler tibiae with BCO.

Molecular & cellular functions	*P*-value	# proteins	Proteins^1^
Organismal survival	3.2 10^–2^–3.210^–2^	26	ACTN4, COL2A1, HMGB1, LUM, PDLM1, SPTAN1, TKT, ALDOC, F13A1, HMOX1, MTAP, PKM, STAT1, UCHL3, APEX1, FLNC, HNRNPA1, MYOF, PLS3, STAT3, APOA1, GNAQ, ITGAV, PCNA, RPL6, TF
Connective tissue development and function	4.4 10^–2^–4.4 10^–2^	5	APOA1, UCHL3, MYOF, STAT3, TKT
Hematological system development and function	4.4 10^–2^–4.4 10^–2^	9	AOPA1, STAT1, CTSZ, HMGB1, PKM, STAT3, LUM, MBD5, HMOX1
Hematopoiesis	4.4 10^–2^–4.4 10^–2^	5	HMGB1, STAT3, LUM, MBD5, STAT1
Immune cell trafficking	4.4 10^–2^–4.4 10^–2^	5	APOA1, STAT1, CTSZ, HMGB1, PKM

^1^ACTN4, Actinin alpha 4; ALDOC, Aldolase, fructose-bisphosphate C; APEX1, apurinic/apyrimidinic endodeoxyribonuclease 1; APOA1, apolipoprotein A1; COL2A1, Collagen type II alpha 1 chain; CTSZ, Cathepsin Z; F13A1, coagulation factor XIII A chain; FLNC, filamin C; GNAQ, G protein subunit alpha q; HMGB1, high mobility group box 1/3; HMOX1, Heme oxygenase 1; HNRNPA1, Heterogeneous nuclear ribonucleoprotein A1; ITGAV, Integrin subunit alpha V; LUM, Lumican; MBD5, Methyl-CpG binding domain protein 5; MTAP, Methylthioadenosine phosphorylase; MYOF, Myoferlin; PCNA, Proliferating cell nuclear antigen; PDLM1, PDZ and LIM domain 1; PKM, Pyruvate kinase M1/2; PLS3, Plastin 3; RPL6, Ribosomal protein L6; SPTAN1, Spectrin, STAT1/3, Signal transducer and activator of transcription 1/3; TF, Transferrin; TKT, Transketolase; UCHL3, Ubiquitin C-terminal hydrolase L3;

**Figure 2:**
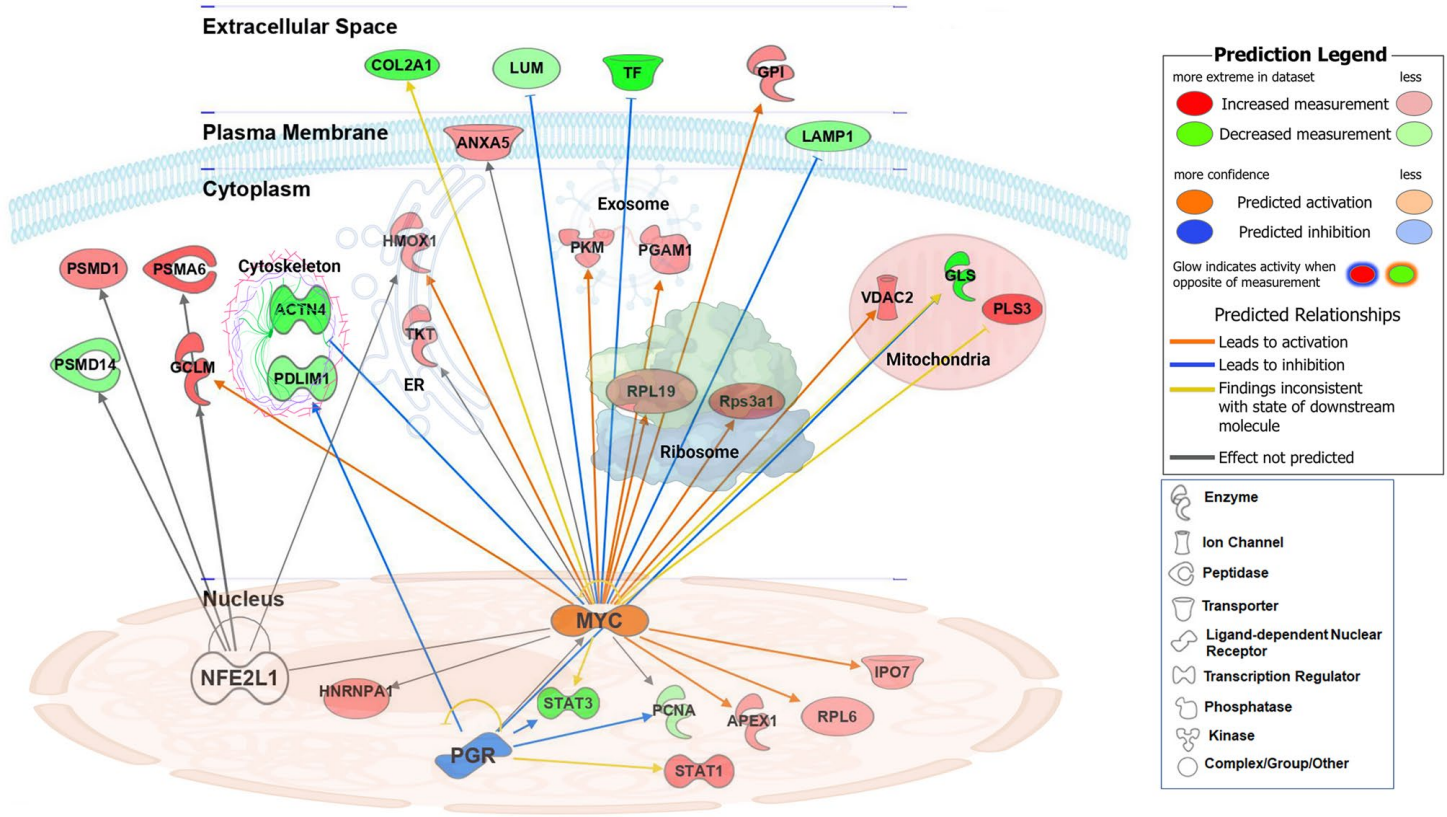
Interconnected proteins and predicted upstream regulators built with IPA program for DE protein data that was determined on BCO-affected tibiae. IPA analysis predicted MYC, PR (PGR), and NRF1 (or NFE2L1) as upstream regulators, which were assigned as inhibited or activated according to Z-score. ACTN4, α-actinin 4; ANXA5, annexin A5; APEX1, Apurinic/Apyrimidinic Endodeoxyribonuclease 1; Col2A1; Collagen Type II Alpha 1 Chain ; GCLM; Glutamate-Cysteine Ligase Modifier Subunit ; GLS; Glutaminase; HMOX1; Heme Oxygenase 1; HNRNPA1; Heterogeneous Nuclear Ribonucleoprotein A1; IPO7; Importin 7; LAMP1; Lysosomal Associated Membrane Protein 1; LUM; Lumican; MYC; Myelocytomatosis Oncogene; NFE2L1; NFE2 Like BZIP Transcription Factor 1; PCNA; Proliferating Cell Nuclear Antigen; PGAM1; Phosphoglycerate Mutase 1; PGR (PR); progesterone receptor; PKM; Pyruvate Kinase M1/2; PLS3; plastin 3; PSMA; Proteasome 20S Subunit Alpha 6; PSMD; Proteasome 26S Subunit, Non-ATPase 1; RPL; Ribosomal Protein L6; RPS; ribosomal protein S3A1; STAT; Signal Transducer And Activator Of Transcription; TF; Transferrin; VDAC2, voltage-dependent anion channel 2.

**Figure 3:**
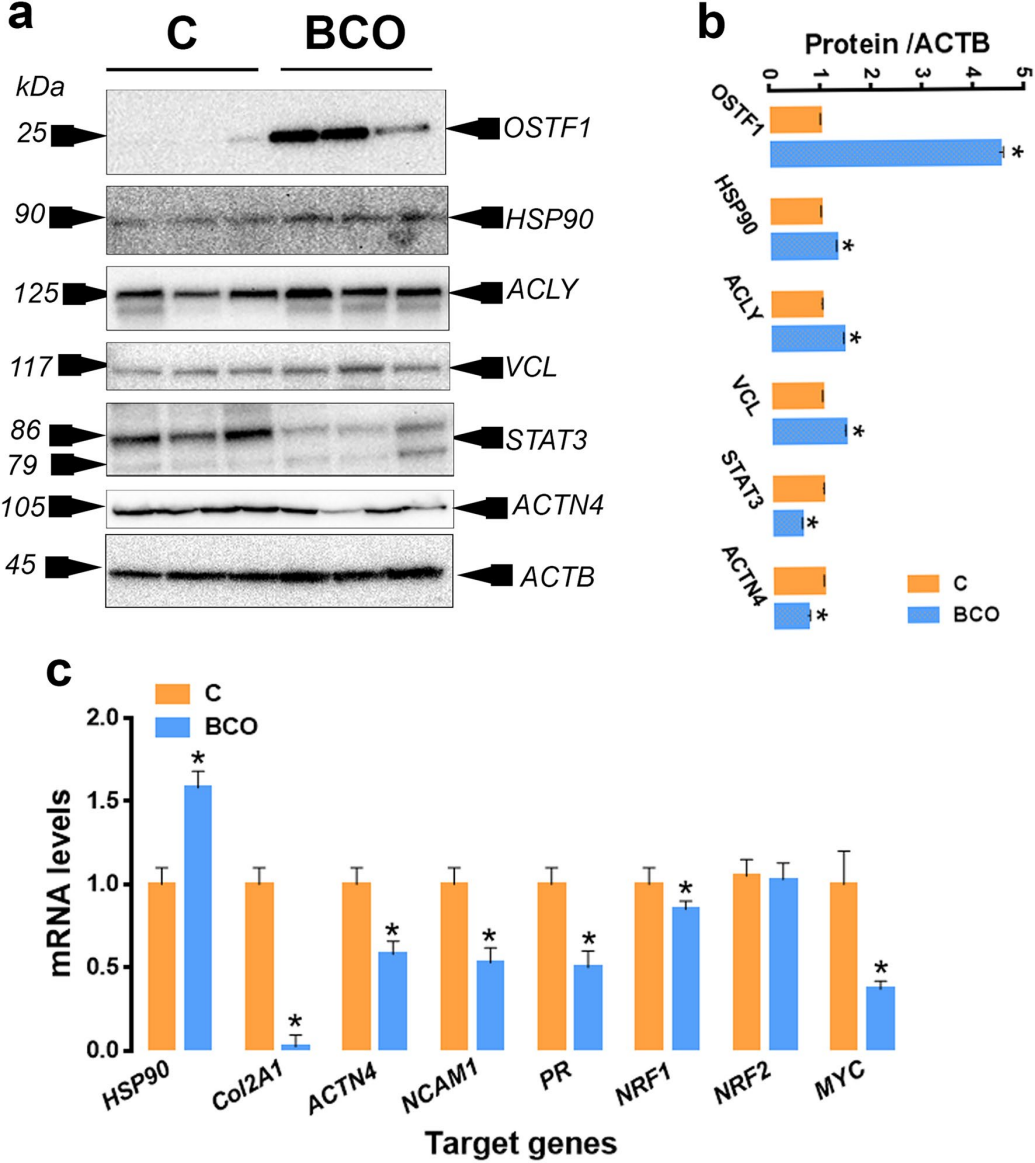
Validation of selected protein-encoding genes. Protein expression was determined by Western blot (**a**, **b**) and mRNA abundances were measured by qPCR and 2^-ΔΔCT^ method^23^. Data are mean ± SEM and * indicates a significant difference at *P* < 0.05. ACLY, ATP citrate lyase; ACTB, β actin; ACTN4, α-actinin 4; Col2A1, Collagen Type II Alpha 1 Chain; HSP90, heat shock protein 90; MYC, myelocytomatosis oncogene; NCAM1, Neural Cell Adhesion Molecule 1; NRF1/2, erythroid 2-related factor 1/2; OSTF1, Osteoclast-stimulating factor-1; STAT3, Signal Transducer And Activator Of Transcription 3; VCL, vinculin.

Figure 2 provides a visual summary of the three IPA-predicted upstream regulators. The top predicted activated upstream transcription regulator in the BCO tibiae was the myelocytomatosis oncogene (MYC) with a computed activation z-score of 2.121, overlap *P*-value of 3.09 × 10^–2^, and with 15 of 23 proteins have measurement direction consistent with MYC activation. However, the progesterone receptor (PR) was significantly enriched by IPA in the THN and was predicted as an inhibitor upstream regulator (z-score −2.0, overlap *P*-value 4.44 × 10^–2^), with 4 of 5 proteins have measurement direction consistent with PR inhibition. The nuclear factor erythroid 2 like 1 (NRF1 or NFE2L1) was also predicted by IPA to be an upstream regulator (Overlap *P*-value 4.44 × 10^–2^), however its activation state was not determined. Validation studies on protein expression profile are more challenging as they rely on availability of antibodies that cross-react with chickens. Here, by using immunoblot, we were able to validate the upregulation of the osteoblast stimulating factor 1 (OSTF1, 4.52-fold change, *P* = 0.0002), heat-shock protein 90 (HSP90, 1.30-fold change, *P* < 0.0001), ATP citrate lyase (ACLY, 1.42-fold change, *P* < 0.0001), and vinculin (VCL, 1.44-fold change, *P* = 0.0005), and the down regulation of STAT3 (0.58-fold change, *P* < 0.0001) and ACTN4 (0.68-fold change, *P* < 0.05, Fig. 3), in BCO-affected compared to healthy-birds, which is in agreement with the LC–MS/MS data. As most of the DE markers are protein-encoding genes, we next determined the mRNA abundances of few selected genes. Real-time qPCR analysis confirmed the up regulation of *HSP90* and the down regulation of *Col2A*1, *ACTN4*, *NCAM1*, and *PR*. Whilst qPCR determined the regulation direction (down regulation) of NRF1, it showed contrary to IPA prediction a down regulation of MYC gene expression (Figs. 2 and 3).

## Discussion

Bacterial chondronecrosis with osteomyelitis is a significant health, welfare, and economic concern in the commercial broiler industry^2^, yet its underlying molecular mechanisms are not fully defined. In the present study, we used the well-established wire-flooring model that is reliable and reproducibly triggers a high incidence of BCO lesions that are similar to BCO-associated lesions observed in commercial flocks^10^. To gain large-scale in-depth knowledge, we used high throughput LC–MS/MS screening and comparative proteomics analysis, which provides a powerful tool to detect differentially or uniquely expressed proteins and their dynamic changes in a particular condition^24,25^. Previous studies have described differential plasma proteomic profile between healthy and spontaneously- or glucocorticoid-induced femoral head necrosis-affected birds^26,27^, and reported potential systemic (serum) biomarkers. In the current study, which constitutes the first to the best of our knowledge, we identified 222 locally (tibial) differentially expressed (DE) proteins, with 158 up- and 64 down-regulated proteins, in tibia of BCO compared to healthy chickens^28^.

The DE proteins were mapped to the reference pathway in IPA knowledge database (QIAGEN Inc., https://digitalinsights.qiagen.com/IPA)^19^ to derive biological insights and identify significantly enriched metabolic and/or signal transduction pathways. IPA identified significant top canonical pathways, including HD disease, ARE-mediated mRNA degradation, protein ubiquitination, hepatic fibrosis signaling, and glycolysis pathways, with several common and overlapping proteins. Based on the IPA core analysis, most of these proteins are involved in several disorders such as bone injury and abnormality, connective tissue disorders and inflammatory diseases. The top physiological functions depicted by IPA include organismal survival, connective tissue development, and immune cell trafficking, which are not surprising.

For instance, among these proteins, proteasomes (PSMA, PSMD, PSME), a multicatalytic proteinase complex, which are ubiquitously distributed in eukaryotic cells to cleave peptides in an ATP/ubiquitin-dependent (non-lysosomal) process^29^, are found in the first three top canonical pathways. They have been shown to be expressed in bone and involved in bone remodeling, resorption, and formation^30–32^. Moreover, infection of human endothelial (HUVEC) cells with *Staphylococcus aureus* (also the leading cause of BCO)^3^ has been shown to induce proteasome subunits at 16 h post-infection^33,34^. Immune and pro-inflammatory pathways have been found to be dependent on both proteasomal activity and ubiquitylation^35^, and our previous studies have shown that pro-inflammatory cytokines, such as IL-1β and TNFα, were induced in BCO-affected chickens at local and systemic levels^36^. This proteasome-induced inflammation is probably mediated through NF-κB and MAPK pathways that merit future investigations^37^. Additionally, proteasome inhibition by NIC-0102 has been reported to specifically prevent NLRP3 inflammasome activation, which has been shown to be induced in bone of BCO-affected chickens and in *Staphylococcus*-infected osteoblasts^21^.

The second group of biomarkers that proteomics analysis found with increased expression in the BCO group are molecular chaperones and stress proteins, including heat shock protein 90 (HSP 90), which also has been confirmed by qPCR and immunoblot analyses. Besides their classical roles as molecular chaperones and housekeepers (folding/unfolding, assembly/disassembly), HSPs are now understood to play a pivotal role in many cellular processes including transport and trafficking, protein degradation, and cell signaling^38^. Depending on the physiological context, these HSPs can be immune-stimulatory or immunosuppressive^39,40^. It has been shown that, after trauma or exposure to bacteria, cells express high levels of HSPs^41^, which in turn lead to cytokine transcription and release^42,43^. Indeed, Takahashi et al.^44^ have reported high levels of HSP70 and cytokines in articular cartilage and suggested a key role for HSPs in early stage of osteoarthritis in both rodents and humans. Moreover, recent studies proposed a role for HSP90 in chondrocyte biology and cartilage breakdown^45,46^. Tribelli et al.^47^, on the other hand, demonstrated that *Staphylococcus aureu*s infection triggers human host primary keratinocyte and HaCaT cell line invasion through HSP90. Consistent with these findings and as mentioned above, the mRNA abundances of pro-inflammatory cytokine IL-1β and TNFα were also higher in the tibiae and femur of BCO compared to the healthy group. As a number of reports have shown that cytokines can stimulate HSP expression^48^, the cause-effect relationship between cytokines and HSP90 in BCO pathogenesis merit further in depth investigations.

The data from our proteomics, immunoblot and qPCR analyses agreed in denoting high levels of osteoclast-stimulating factor 1 (OSTF1), a protein known to activate osteoclasts and modulate trabecular bone remodeling^49^. OSTF1 was first described as an intracellular SH3-domain containing protein produced by osteoclasts that indirectly induces osteoclast formation and bone resorption^49^. Under normal physiological conditions, bone mass and structure homeostasis are maintained by constant bone remodeling with balanced bone formation by osteoblasts and bone resorption by osteoclasts^50,51^. Interestingly, Wideman group has previously found, in BCO model, a high osteoclastic activity^52^, which dissolve bone mineral by massive acid secretion and production of specialized proteinases that degrade the organic matrix, mainly type I collagen and erode the trabecular bone^53^. The increased levels of cathepsins here, which are responsible for the degradation of type I collagen in osteoclast-mediated bone resorption reinforce the aforesaid data^54^. Vermeren and co-workers^55^ reported that OSTF1 knockout mice suffer from a mild form of osteopetrosis, which caused by an increase in trabecular bone. In support of the abovementioned results, we reported here a reduced expression of Col2A1, at mRNA and protein levels, in BCO compared to control birds, which confirm a degradation status of collagen matrix and excessive bone resorption caused by exaggerated osteoclast activity-induced OSTF1 overexpression. Heretofore, molecular defects in the Col2A1 gene has been found to lead to low bone mass, bone deformity and fragility, and increased fracture incidence^56^, and thereby resulted in skeletal disorders such as skeletal dysplasia, achondrogenesis, stickler syndrome, and osteoarthritis^57^.

Parallel to Col2A1 down regulation, several other proteins (n = 20 from IPA top analysis-ready molecules) were found to be decreased (−2.3 < Expr Log Ratio) or increased (Expr Log Ratio > 2.4). Of particular interest, ovotransferrin (OTF), an 82-kDa glycoprotein and a member of transferrin family^58^, was significantly decreased in BCO. OTF and its receptor were found to be expressed in chicken bone and play key roles in bone formation^59^. In addition, mechanistically disruption of transferrin system altered iron uptake, heme biosynthesis, and bone homeostasis through glycolysis- and mitochondrial oxidative phosphorylation-dysmetabolisms^60^, both of which were delineated by proteomics and IPA analysis. In line with this, the ATPase H^+^ transporting V1 subunits (ATP6V1D/ATP6V1E1) that play critical roles in iron homeostasis and ATP synthesis were dysregulated. Similarly, the mitochondrial voltage dependent anion channels (VDAC2), glutaminase (GLS), and plastin 3 (also known as fimbrin, PLS3) expressions were dysregulated, indicating a mitochondrial dysfunction in bone of BCO birds, which has been previously reported by our group^61^. VDAC2 has been reported to play key roles in ADP-dependent mitochondrial bioenergetics^62^. PLS3, a member of actin-binding and bundling protein family, plays a pivotal role in actin cytoskeleton and in mitochondrial motility and function^63^. GLS, a key mitochondrial enzyme that catalyzes the deamidation of glutamine^64^, plays essential roles in oxidative phosphorylation, glutathione synthesis, and cellular redox homeostasis^65^. The decreased expression of glutathione S-transferase 1 (GSTT1), a multifunctional enzyme involved in oxidative stress, along with the antioxidant peroxiredoxin 6, in our experimental conditions supported the aforementioned data and indicated a potential accumulation of mitochondrial ROS in BCO-affected bone^66,67^.

One of the best-characterized pathways leading to cell death, a hallmark of BCO bone, involves mitochondria through outer membrane permeability, inner membrane potential changes, as well as elevated ROS production^68^. All the above DE mitochondrial markers (VDAC2, GLS, PLS3) have been reported to be involved in cell death^69–74^. Although further mechanistic studies are warranted, as bone resorption requires rapid cytoskeletal reorganization (sealing zone consisting of actin filament core surrounded by actin-binding proteins), we postulate that bacterial infection in BCO pathology dysregulates this actin cytoskeleton. This is supported, here, by dysregulation of PLS3, actin α1/γ1, and actin-binding proteins (ABPs) such as filamins (actin branching)^75^, tropomyosins (actin stabilizing)^76^, myosins (actin filament contraction and bundling)^77^**,** talin1^78^, integrins (subunit αV, and β)^79^, actinins (ACTN1/4, actin cross-linking proteins)^80^, annexins^81^, fibronectin 1^82^, hemopexin (heme scavenger)^83^, nebulin^84^, radixin^85^, stomatin^86^, vinculin^87^, vitronectin^88^, lamin^89^, scinderin (calcium-dependent actin filament-serving protein)^90^, and actin related protein 2/3 complex subunit 1B (ARPC1B). A number of studies have demonstrated a role for the actin cytoskeleton and several ABPs in triggering apoptosis upstream of caspases^91,92^, which has been shown by our group to be involved in bone attrition and osteoblast death^21^. This dysregulation of actin cytoskeleton complex alters the opening of VDAC, dysregulates the mitochondrial membrane permeabilization, depolarization, and integrity, and thereby leads to key apoptotic process via increased ROS production and oxidative stress^93,94^.

In addition to the mitochondria, ribosomes, nucleus, and nucleolus are central hubs for stress sensors^95^. Proteomic analysis identified here several DE ribosomal proteins (RPs), including RPL6, RPL7a, RPL19, RPL70, RPS2, RPS3, RPS3A1, RPSA, and RRBP1. Although RPs are well established as the basic building blocks in the ribosome assembly and biogenesis, as well as protein translation and synthesis^96^, there is increasing evidence indicating that RPs play critical roles in normal cell physiology, cellular response to stress, insults, and diseases^97–99^. In fact, it has been demonstrated that RPs have extra-ribosomal functions, including DNA repair, cell- cycle arrest, and apoptosis^100–103^. Specifically, RPS2 and RPL7A have been shown to be targets for mir-320a and to be involved in cartilage degradation^104^ and osteoporosis^105^. RPL19 and RPS3A1 have been found to be targets for mir-16-5p and to be involved in osteoarthritis^106^, osteoclastogenesis^107^, and rheumatoid arthritis^108^. In addition of being a RP, RPS3 has been shown to be a DNA repair endonuclease that is involved in apoptosis^109^. Together our proteomic data unveiled for the first time a potential key watchguard role of RPs, ribosomal stress, and ribosomopathy in BCO pathogenesis, however more questions related to whether (1) these RPs are free or membrane-bound? (2) These RPs are nucleolar or mitochondrial? (3) The RP perturbation is a consequence or an associated feature of BCO? And the nature of downstream cascades of these RPs beg to be answered. It is possible that RPs are directly or indirectly involved in various downstream signaling pathways, including RP-MDM2-P53 signaling^110^, NF-κB-Gadd45β pathway^111^, and/or proteasome-ubiquitin pathways^112,113^, which has been pinpointed by LC–MS/MS analysis. For instance, it has been shown that the binding of RPL26 to MDM2 promote the ubiquitination and proteasomal degradation of RPL26, which inhibits the P53 protein synthesis through the disruption of PRL26-P53 mRNA association^114^. Furthermore, the RPS7 was found to be a substrate for MDM2-meditaed ubiquitination, and the RPS7-ubiquitin fusion protein selectively inhibits MDM2-mediated P53 degradation and induces apoptosis^115^.

As disruption of ribosome biogenesis leads to nucleolar stress, proteomics and IPA analyses identified several DE-nuclear proteins between BCO-affected and healthy birds. Signal transducer and activator of transcription (STAT1 and STAT3) were oppositely dysregulated in tibiae of BCO-affected compared to healthy birds. These proteins belong to JAK-STAT family that contains at least seven members encoded by distinct genes, which are both signal transducers and transcription factors^116^. It has been shown that the STAT proteins were differentially activated in a context-dependent manner in response to various stimuli^117^. The down regulation of STAT3 in our experimental conditions supports its role in apoptosis and bone attrition^118^. Davidson and colleagues^119^ have shown that loss of STAT3 has a detrimental effect on osteoclast and bone structure. Boone et al.^120^ have reported an association between STAT3 deficiency and child hip osteonecrosis. Zhou et al.^121^ demonstrated a critical role for STAT3 in skeletal development and bone homeostasis. Using hematopoietic cell-specific STAT3 knockout mice, Zhang et al.^122^ have reported an accelerated osteoporosis with increased osteoclastogenesis. STAT1, on the contrary, has been shown when it is overexpressed, to enhance apoptotic cell death in cardiac myocytes exposed to ischemia-reperfusion^123^, however overexpression of STAT3 reduced STAT1-induced cell death. Furthermore, increasing number of studies confirmed that STAT1 and STAT3 have opposing actions on apoptotic cell death in various cell types^124^, via antagonistic effects on promoters of genes encoding anti-apoptotic BCL-2 and BCL-X proteins^125^. Kim et al.^126^ showed that STAT1^-/-^ mice exhibited excessive osteoclastogenesis. Moreover, Xiao et al.^127^ have reported that STAT1 control bone formation via FGF signaling (Supplementary Fig. S1).

Curiously, IPA predicted PR, NRF1, and MYC as potential upstream regulators involved in BCO pathogenesis. Although the opposite regulation was detected by qPCR, the role of MYC in the apoptotic pathways is confounding and not fully understood^128–130^. MYC is a proto-oncogene, which encodes for a nuclear phosphoprotein that plays a key role in cellular transformation and apoptosis^129,131,132^. The target genes for MYC approached 4000 in human, that are involved in various cellular processes, including cell cycle, survival, protein synthesis, cell adhesion, cytoskeleton, and metabolism^133,134^. It is worth mentioning that HSP90^135^, OSTF1^136^, VDAC2^137^, RPs^138,139^, mitochondrial genes^140^, Col2A1^141^, and cytoskeleton-associated proteins^142–144^, are all targets for MYC. Of particular interest, MYC was found to be expressed in bone and required for osteoclast differentiation^145^. Moreover, MYC dysregulation has been shown to affect collagen and induce apoptosis and cartilage degeneration^141^.

Although its function remains elusive, epidemiological, clinical, and experimental data indicated that progesterone is active in bone metabolism and that PRs are expressed in human osteoblast^146^. Turner’s group has reported a high bone mass phenotype in global-PR knockout mice, which appeared to result from a reduced bone resorption rate in male, and a greater bone formation rate in female^147^. Wang and co-workers demonstrated that progesterone suppressed murine osteoblast MC3T3-E1 apoptosis via activation of PR and inhibition of caspase 3 and 9 activities, as well as cytochrome c release^148^. This effect was reversed by PR antagonist which supports IPA-predicted and qPCR-confirmed data here, that down regulation of PR might induce bone attrition in BCO-affected tibiae. Although the upstream mechanism by which the *Staphylococcus* infection down regulates PR is not known, the down-stream pathways mediated by PR is likely involve MYC^149^, Bcl-2^150^, STAT1^151^, MAPK^152^, and/or mTOR^153^.mTOR complexes are well established to regulate, among other things, protein synthesis and cell survival pathways^154,155^. Recently, Manning’s group has uncovered a surprising new function of mTOR in increasing cellular proteasome via NRF1 induction^156,157^, which has been predicted here by IPA analysis and confirmed by qPCR. NRF1, which is also known as NFE2L1/LCRF1/TCF11, is a member of the CNC subfamily of basic-leucine zipper (bZIP) transcription factors^158^. There are two NRF1 isoforms; a 120 kDa isoform localized primarily in the ER as an integral membrane protein, whereas the 65 kDa isoform is nuclear^159^. Binding-site selection experiments have shown that NRF1 binds preferentially to a consensus sequence that is identical to the antioxidant response element (ARE)^160^, which regulates numerous oxidative stress-related genes^161^. In furtherance of our data, osteoblast-specific NRF1-knockout mice have reduced bone mineral content and bone area^162^.

In summary, this is the first study using high throughput analysis in combination with bioinformatics tools to evaluate tibia proteome in BCO-affected and healthy broilers. Several DE proteins, protein interaction networks, disease-and function-based networks, canonical pathways, and upstream regulator were identified. We validated a panel of protein/gene candidates that following further mechanistic and functional studies may be potential biomarkers for BCO pathogenesis.

## Supplementary Information

Supplementary Information 1.

## Data Availability

The proteomic datasets generated during the current study are available in PRIDE database (EMBL-EBI ProteomeXchange, PRIDE database, https://doi.org/10.6019/PXD029085, with the accession PXD029085). To access please use the following ID: dridi@uark.edu and the PW: DrR76fJD.
