# Supplementary figures and images for: Comparative- and network-based proteomic analysis of bacterial chondronecrosis with osteomyelitis lesions in broiler’s proximal tibiae identifies new molecular signatures of lameness
Source: Sci Rep. 2023 Apr 12;13:5947. doi: 10.1038/s41598-023-33060-y (PMC10097873; doi:10.1038/s41598-023-33060-y)

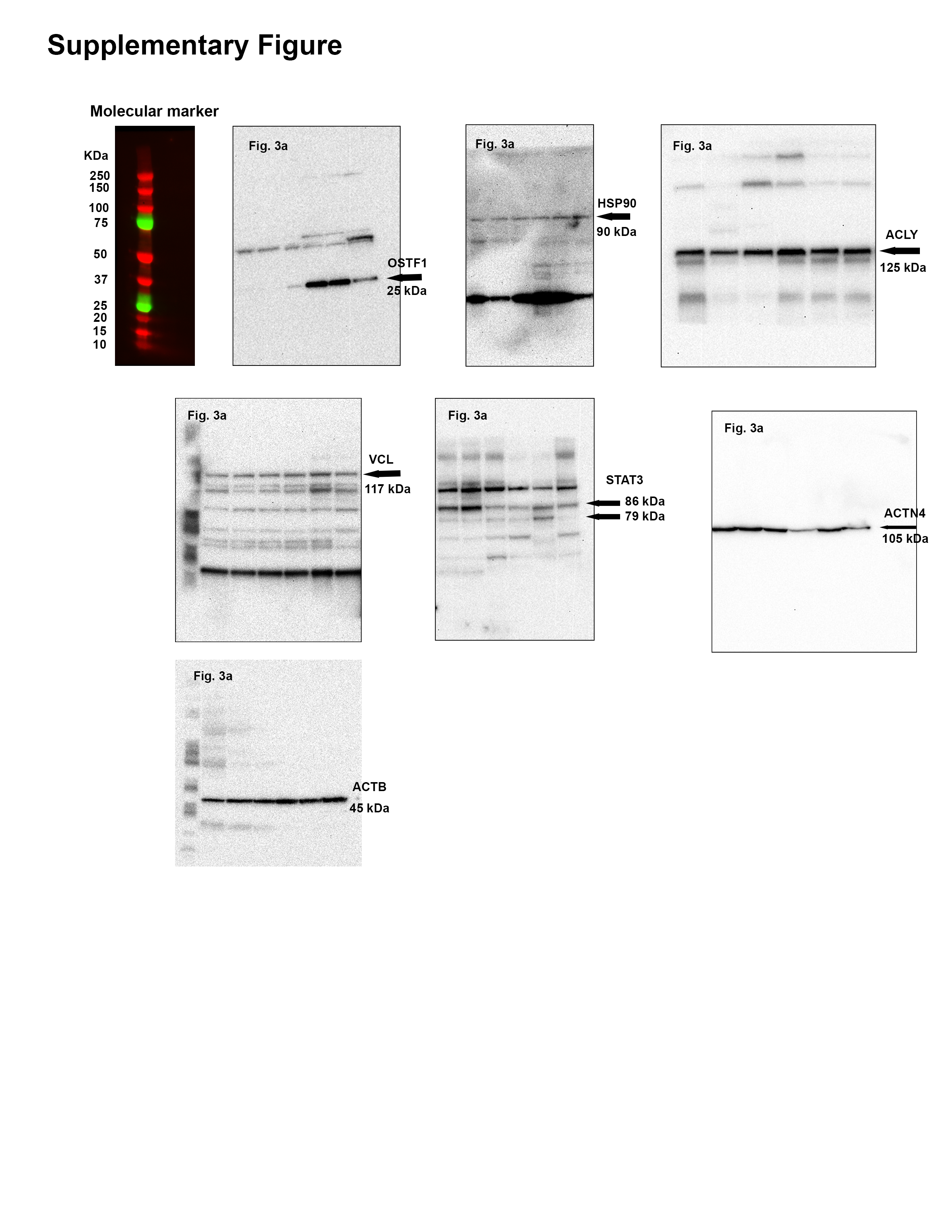

Supplement: Supplementary file 1 — Supplementary Information 1. [file 41598_2023_33060_MOESM1_ESM.tif]
